# Peptide-Based Subunit Vaccine against Hookworm Infection

**DOI:** 10.1371/journal.pone.0046870

**Published:** 2012-10-03

**Authors:** Mariusz Skwarczynski, Annette M. Dougall, Makan Khoshnejad, Saranya Chandrudu, Mark S. Pearson, Alex Loukas, Istvan Toth

**Affiliations:** 1 The University of Queensland, School of Chemistry and Molecular Biosciences, St. Lucia, Queensland, Australia; 2 Centre for Biodiscovery and Molecular Development of Therapeutics, Queensland Tropical Health Alliance, James Cook University, Cairns, Queensland, Australia; 3 The University of Queensland, School of Pharmacy, Wooloongabba, Queensland, Australia; The George Washington University Medical Center, United States of America

## Abstract

Hookworms infect more people than HIV and malaria combined, predominantly in third world countries. Treatment of infection with chemotherapy can have limited efficacy and re-infections after treatment are common. Heavy infection often leads to debilitating diseases. All these factors suggest an urgent need for development of vaccine. In an attempt to develop a vaccine targeting the major human hookworm, *Necator americanus*, a B-cell peptide epitope was chosen from the apical enzyme in the hemoglobin digestion cascade, the aspartic protease *Na*-APR-1. The A_291_Y alpha helical epitope is known to induce neutralizing antibodies that inhibit the enzymatic activity of *Na-*APR-1, thus reducing the capacity for hookworms to digest hemoglobin and obtain nutrients. A_291_Y was engineered such that it was flanked on both termini by a coil-promoting sequence to maintain native conformation, and subsequently incorporated into a Lipid Core Peptide (LCP) self-adjuvanting system. While A_291_Y alone or the chimeric epitope with or without Freund’s adjuvants induced negligible IgG responses, the LCP construct incorporating the chimeric peptide induced a strong IgG response in mice. Antibodies produced were able to bind to and completely inhibit the enzymatic activity of *Na*-APR-1. The results presented show that the new chimeric LCP construct can induce effective enzyme-neutralising antibodies in mice, without the help of any additional toxic adjuvants. This approach offers promise for the development of vaccines against helminth parasites of humans and their livestock and companion animals.

## Introduction

Hookworm infection causes one of the world’s most debilitating neglected tropical diseases. The human hookworm (*Necator americanus*) infects over 700 million people worldwide, predominantly in indigent rural and tropical regions [1]. Chronic infection results in long-term pathological consequences primarily due to ongoing intestinal blood loss resulting from the feeding activities of these hematophagous parasites. Heavy infection leads to iron-deficiency anemia and can manifest as impaired neurological and intellectual functioning in children, reduced work capacity in adults, and severe adverse outcomes in pregnancy [2]. Those most vulnerable to the harmful effects of hookworm include children and pregnant women, who are unable to tolerate the chronic blood loss and iron deficiency anemia due to their lower iron reserves [3]. These factors have significant influence on current and future productivity and economic well being of infected populations. Benzimidazole drugs are commonly used for the treatment and eliminate adult parasites. However, chemotherapy has limited efficacy and reinfection after treatment is common [4], [5]. The problems with drug effectiveness and the looming threat of drug resistance suggest that alternatives to mass drug administration are urgently needed. Development of a vaccine that would prevent the acquisition of moderate or heavy intensity hookworm infection would be a major advance in reducing the morbidity caused by this parasite [6], [7]. Currently, there is no human hookworm vaccine on the market or in advanced clinical trials.

Hookworms obtain their nourishment primarily by ingesting blood and digesting the hemoglobin and serum proteins released from lysed erythrocytes. *Na*-APR-1 is a cathepsin D aspartic protease derived from the gut of adult *N. americanus* where it initiates the hemoglobin digestive cascade [8], [9]. Therefore, blocking the catalytic activity of *Na*-APR-1 via the induction of neutralizing antibodies should result in starving and ultimately killing of the parasite. Indeed, it was demonstrated that APR-1 could be used as an efficacious hookworm vaccine antigen against *A. caninum* in dogs. Vaccination with the recombinant *N. americanus* or *A. caninum* enzymes induced antibodies that bound to the gut of the parasite and neutralized the enzymatic activity of the protease *in vitro*. When vaccinated dogs were then challenged with hookworm larvae they had significantly diminished adult parasite burdens and a reduction in blood loss was observed [8], [10]. However, production of APR-1 at a commercial scale has proven to be challenging due to protein aggregation and low manufacturing yield obtained from eukaryotic expression systems. Previously, the immunogenic peptide epitope from *N. americanus* APR-1 (A_291_Y, AGPKAQVEAIQKY) was shown to induce the production of neutralizing antibodies *in vivo*. These antibodies were able to inhibit enzymatic activity of APR-1 against synthetic peptide and natural protein substrates [11].

Peptide antigens are not immunogenic by themselves and require appropriate delivery systems and strong, often toxic, adjuvants to stimulate desired immune responses [12]. For example, in the above-mentioned study the use of toxic complete Freund’s adjuvant composed of inactivated and dried mycobacteria was necessary to stimulate immune responses against the A_291_Y epitope. To avoid this problem, lipidation of peptides emerged as a promising strategy for delivery of peptide subunit vaccines. The self-adjuvanting Lipid Core Peptide (LCP) delivery system has a demonstrated ability to induce strong immune responses against the incorporated peptide-epitopes without the help of external adjuvants, and is considered to be a capable platform for development of human vaccines [13], [14].

In this paper, we have designed, synthesized and characterized a short series of A_291_Y conjugates. We have used the coil-promoting sequence from the yeast GCN4 protein to induce native helical A_291_Y epitope conformation [11], [13], [15], [16]. Subsequently, chimeric and parent A_291_Y epitopes were incorporated into the LCP delivery system. The various LCP constructs induced variable IgG responses in mice, nonetheless these antibodies were able to bind to and completely inhibit the enzymatic activity of *Na*-APR-1. The results presented show that the new chimeric epitope can be presented as an LCP construct to induce an effective enzyme-neutralizing response without the help of any additional adjuvants and delivery systems. This approach offers promise for the development of vaccines against helminth parasites of humans and their livestock and companion animals.

## Materials and Methods

### I. Materials

Protected L-amino acids were obtained from Novabiochem (Laufelfingen, Switzerland) and Mimotopes (Melbourne, Australia). pMBHA resin was purchased from Peptides International Inc. (Kentucky, USA). 1-(1H-benzotriazol-1-yl)-1,1,3,3-tetramethyluronium hexafluorophosphate (HBTU) was purchased from Mimotopes. HPLC grade acetonitrile and *N*,*N*-dimethylformamide were obtained from Ajax Finechem (Sydney, Australia). Trifluoroacetic acid (TFA) was obtained from Merck (Kilsyth, Australia). All other reagents were obtained from Sigma-Aldrich (Castle Hill, NSW, Australia). Microwave assisted Fmoc SPPS was carried out by using a SPS mode on CEM Discovery reactor (CME Corporation, Matthews, NC, USA). An AKel-F HF apparatus (Peptide Institute, Osaka, Japan) was used for HF cleavage. ESI-MS was performed on a Perkin-Elmer-Sciex API3000 instrument with Analyst 1.4 software (Applied Biosystems/MDS Sciex, Toronto, Canada). Analytical RP-HPLC was performed on an Agilent instrument with a 1.0 mL/min flow rate and detection at 214 nm. Separation was achieved by running gradient mode of 0–100% solvent B over 40 min with solvent A (0.1% TFA/H_2_O) and two kind of solvent B (B1∶90% MeCN/0.1% TFA/H_2_O or B2∶90% MeOH/0.1% TFA/H_2_O) on either a Vydac analytical C4 column (214TP54; 10 µm, 4.6×250 mm) or a Vydac analytical C18 column (218TP54; 10 µm, 4.6×250 mm). Purification was carried out by preparative RP-HPLC using a Waters Delta 600 system with a 10.0 mL/min flow rate. Compounds were detected at 230 nm. Separations were performed with solvent A and solvent B1 on either a Vydac preparative C4 column (214TP1022; 10 mm, 22×250 mm) or a Vydac preparative C18 column (218TP1022; 10 mm, 22×250 mm). CD spectra were measured on a JASCO J-710 spectropolarimeter (Tokyo, Japan) using a quartz cuvette of 1 mm path length at 23°C. The CD spectra were measured in water with trifluoroethanol (TFE) at concentration of 10% (v/v).

### II. Synthesis of Peptide Epitopes and LCPs

**Figure 1:**
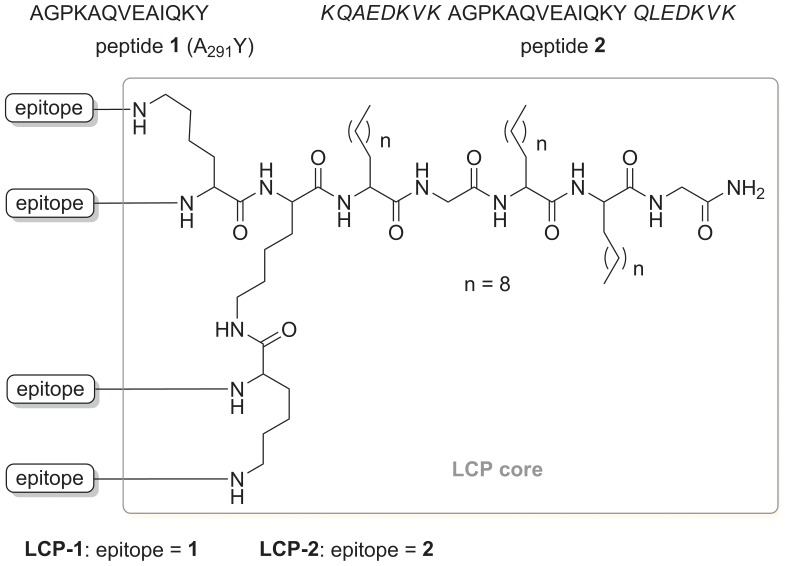
The structure of peptide epitopes (1, 2) and LCP constructs (LCP-1 and LCP-2). Peptide epitope **2** retains native epitope sequence **1** (regular fonts) flanked by helicity inducing sequence from GCN4 protein (italic fonts).

Peptides **1** and **2** (Figure 1) were synthesized using manual SPPS at 0.1 mmol scale with pMBHA (0.45 mmol NH_2_/g) resin. The synthesis was carried out as previously reported [17], [18], [19]. Shortly thereafter, Boc-deprotection was performed with neat TFA (2×1 min), followed by a 1 min DMF flow wash, and a 30 min coupling with pre-activated Boc-protected amino acid (1 hour in the case of lipoamino acid (2-(*R*/*S*)-[(tert-butoxycarbonyl)amino]-dodecanoic acid)) [17], [20]. Amino acid pre-activation was achieved by dissolving amino acids (4.2 equiv.) in 0.5 M HBTU/DMF solution (4.0 equiv.), and DIPEA (6.2 equiv.). Activation proceeded for 2–3 min, except for lipoamino acids which were pre-activated for 5–10 min. Double coupling was performed for all amino acids.

#### Peptide 1

Yield: 33%. Molecular Weight: 1443.7. [M+1H^+^]^1+^ m/z 1444.7 (calc. 1444.77), [M+2H^+^]^2+^ m/z 722.8 (calc. 722.89). t_R_ = 13.79 (0–100% Solvent B1, 30 min; C18), t_R_ = 21.37 (0–100% Solvent B2, 30 min; C18).

#### Peptide 2

Yield: 28%. Molecular Weight: 3210.7. [M+3H^+^]^3+^ m/z 1071.8 (calc. 1071.25), [M+4H^+^]^4+^ m/z 803.9 (calc. 803.69). t_R_ = 13.8 (0–100% Solvent B1, 30 min; C18), t_R_ = 21.33 (0–100% Solvent B2, 30 min; C18).

**LCP-1** and **LCP-2** (Figure 1) were synthesized using microwave-assisted SPPS at 0.05 mmol scale with pMBHA (0.45 mmol NH_2_/g) resin. Microwave-assisted SPPS was applied with two five minute microwave-assisted couplings (SPS mode, power 20 W, temperature 70°C, ΔT  = 1°C) for each amino acids (4.2 equiv.) using HBTU (4.0 equiv.) and DIPEA (6.2 equiv.) [21]. The lipoamino acids were coupled in the same manner as regular amino acids but with extended (5–10 mins) HBTU/DIPEA pre-activation. N-terminal acetylation, TFA-assisted Boc cleavage and final cleavage from resin was performed as reported previously [17].

#### LCP-1

Yield: 46%. Molecular Weight: 6814.1. [M+5H^+^]^5+^ m/z 1364.3 (calc. 1363.81), [M+6H^+^]^6+^ m/z 1136.8 (calc. 1136.67). t_R_ = 20.7, 20.96, 21.19, 21.66 (0–100% Solvent B1, 30 min; C4), t_R_ = 29.82, 30.39, 30.61, 31.03 (0–100% Solvent B2, 30 min; C4).

#### LCP-2

Yield: 38%. Molecular Weight: 13886.1. [M+11H^+^]^11+^ m/z 1263.1 (calc. 1263.38), [M+12H^+^]^12+^ m/z 1158.4 (calc. 1158.11). t_R_ = 18.39, 18.61 (0–100% Solvent B1, 30 min; C4), t_R_ = 27.20 (0–100% Solvent B2, 30 min; C4).

### II. Toxicological Evaluation

#### Hemolytic assay

**Figure 2:**
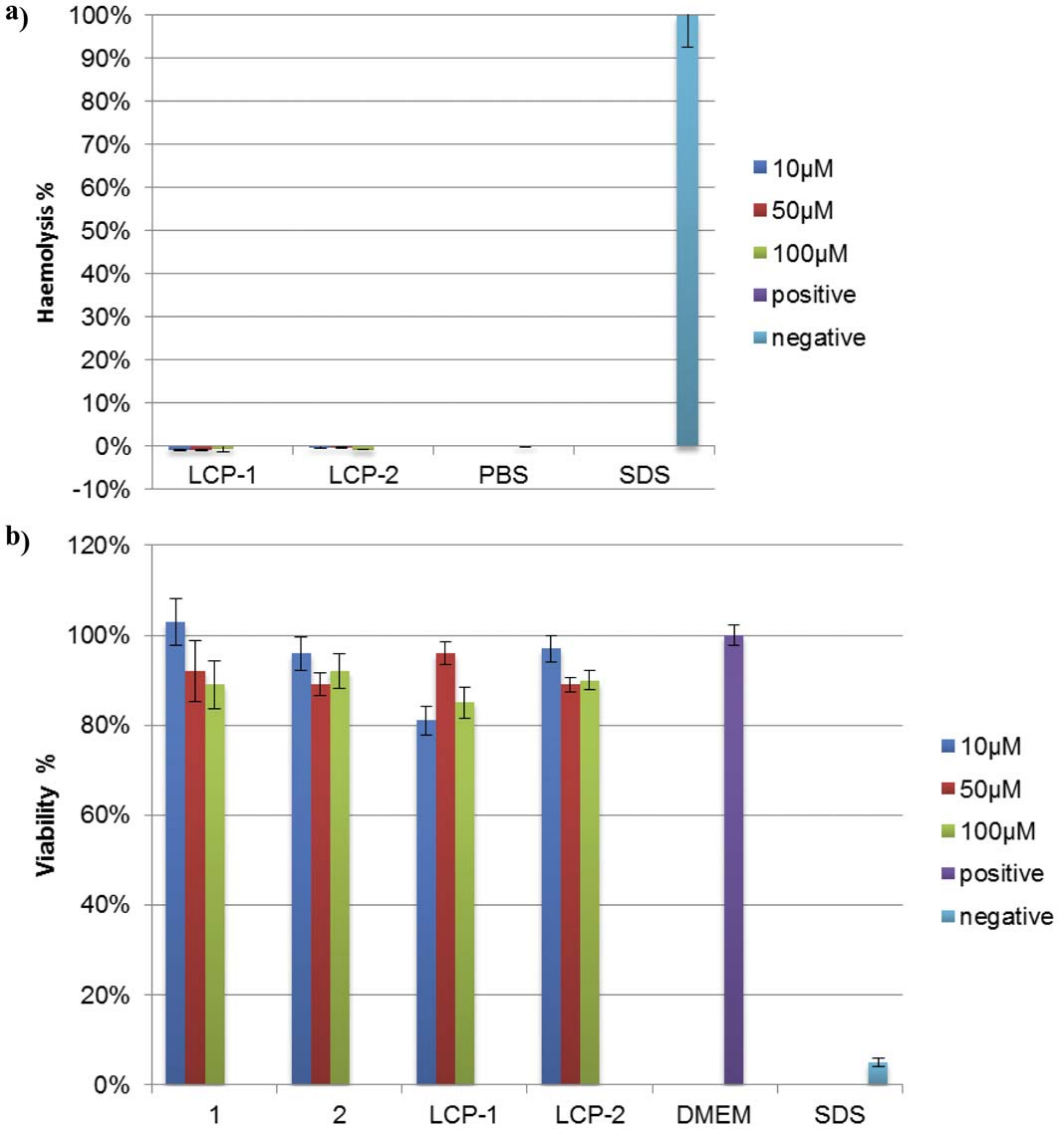
Toxicity of lipid core peptides LCP-1and LCP-2. (a) Haemolytic potential of lipid core peptides (LCP) was measured by comparing the absorbance (540 nm) of blood samples incubated with the LCP vaccine candidates with that of samples incubated with a positive control (SDS, 100% haemolysis) and a negative control (PBS, 0%). Mean and SD of triplicates samples shown. (b) 3-(4,5-Dimethylthiazol-2-yl)-2,5-diphenyltetrazolium bromide cell viability assays in triplicate were performed on the Caco-2 cell line with varying concentrations of compounds (10–100 µM).

Using a standard hemolytic assay, the capacity of LCP compounds (**LCP-1** and **LCP-2**) to induce hemolysis was examined (Figure 2). Blood was collected from a healthy human volunteer with written informed consent (protocol approved by the University of Queensland Ethics Committee, approval number 2009000661). The toxicity of the compounds was tested in three concentrations, (10, 50, and 100 µM) and incubated at 37°C for one hour. SDS was used as the positive control and PBS as negative control. After one hour, the plate was centrifuged at 750× *g* for 15 min and 75 µL of supernatant per well was transferred to a new 96 well plate. The absorbance at 540 nm was recorded by UV spectrometer. The data was calculated according to a standard formula:
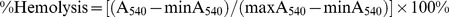
where:

-A_540_ is the average absorption of compound at 540 nm
-minA_540_ is the average absorption of PBS
-maxA_540_ is the average absorption of SDS.

#### MTT assay

The toxicity of the compounds (**1–4**) was examined by MTT assay (Figure 2). The Caco-2 cells were cultivated in a flask with DMEM (Dulbecco’s Modified Eagle Medium) to reach 80% confluence. The cells were split into a 96 well plate (100 µL of cells per well) prior to the MTT test (to let the cells adhere to on the surface). The compounds **1**–**4** were prepared in three concentrations of 10, 50, and 100 µM in PBS. The culture medium was removed and 100 µL of the compound solutions were added to each well. The cells were incubated for 24 hours. After the incubation, the solutions were removed and 20 µL of MTT followed by 80 µL of DMEM medium was added to each well. The plates were incubated for 4 hours, centrifuged at 750× g for 5 mins, and the supernatant discarded. Fifty microliters of DMSO was added to each well to dissolve the purple crystals. The UV absorbance readings were taken at 570 nm wavelength. PBS was used as a blank and SDS, 100% as a negative control. The data was calculated according to a standard formula:
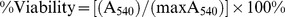
where:

A_540_ is the average absorption of compound at 540 nm
maxA_540_ is the average absorption of DMEM (positive control).

### III. Intraperitoneal Immunization of Mice with Constructs

Groups of four female BALB/c mice (4–6 weeks old) were intraperitoneally injected with 30 µg of peptide 1+Fruend’s adjuvants, peptide 2+Fruend’s adjuvants, LCP-1 or LCP-2 in 200 µL of PBS. All animal protocols used were approved by the James Cook University Ethics Committee (A1484) in accordance with National Health and Medical Research Council (NHMRC) of Australia guidelines. For the Fruend’s adjuvants groups, peptides 1 and 2, 100 µL (30 µg) of construct was mixed with an equal volumes of Freund’s complete adjuvant (CFA) for the first immunization and incomplete adjuvant for the subsequent immunizations (Sigma). Mice were injected 4 times on days 0, 21, 33, and 43. Mice were euthanized on day 70 and the blood collected via cardiac puncture. Sera were separated from clotted blood by centrifugation at 3 000 rpm for 10 min.

In a second experiment, a total of five female BALB/c mice were immunised with 60 µg per immunization of **LCP-2** on days 0, 21, 32, and 35. On day 42 mice were euthanized and the sera collected as above.

### IV. ELISA

Antibody responses to the constructs were measured by ELISA. Peptides or LCPs were coated on 96 well flat bottom ELISA plates (BD) at a concentration of 5 µg/mL in 50 mM sodium carbonate buffer at pH 9.6 and incubated overnight at 4°C. Non-reactive sites were blocked with 3% bovine serum albumin in PBS/0.05% Tween 20 (Sigma) for 1 hr at RT. Individual mouse sera were added in duplicate using 10-fold serial dilutions to plates that were coated with the respective immunogens. Total IgG binding was detected using 1∶2000 sheep anti-mouse IgG (H&L) conjugated to horseradish peroxidase (Chemicon). Bound anti-mouse IgG was detected using TMB single solution (Invitrogen). Plates were measured at 655 nm on a *POLARstar* Omega *microplate reader (BMG Labtek)*. Absorbance was measured at 655 nm and corrected for background using wells that received sera (1∶100) in the absence of peptide or LCP bound to the plate.

### V. Enzyme Neutralisation Assay

The IgG from the two highest responding mice (experiment 2) were bound to protein G sepharose (Millipore) and eluted as previously described [8], [11]. After purification, IgG was concentrated and buffer exchanged into PBS using Nanosep® centrifugal devices (Pall) as per the manufacturer’s protocol. Yeast derived recombinant of *Na*-APR-1 (60 ng) (kindly provided by Dr Bin Zhan, George Washington University) was incubated with 2.5 and 1.25 µg of purified IgG in 50 mmol/L sodium acetate. Reactions were performed in black 384 well plates (Greiner Bio One) in 50 µl; the substrate 7-methoxycoumarin-4-acetyl-GKPILFFRLK(DNP)-d-Arg-amide (MoCAc-GKPILFFRLK) (Sigma) was added to a final concentration of 1.0 mmol/L, and the fluorescence generated (relative to substrate in buffer alone) by substrate hydrolysis was measured as described [8] using a *POLARstar* Omega *microplate reader*. Inhibition of enzymatic activity was analysed as a percentage of the fluorescence generated from an equivalent reaction containing equivalent amounts of control IgG (Millipore) at 2.5 µg and 1.25 µg.

## Results

### I. Synthesis and Characterization

**Figure 3:**
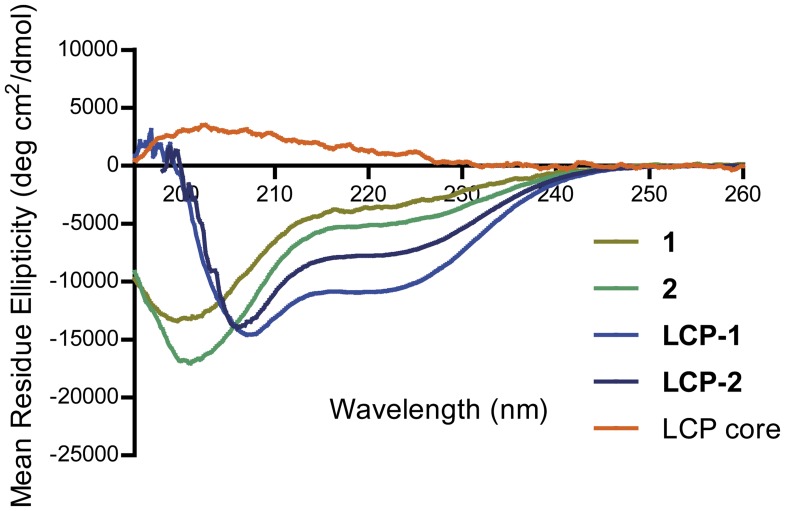
CD spectra of peptides 1, 2, LCP-1 and LCP-2 in the presence of 10% of TFE. Spectra were acquired at 1 nm intervals from 260 nm to 190 nm, were the average of 3 individual scan and are reported in units of mean residue ellipticity [θ].

All compounds were synthesized using the stepwise Boc-SPPS method in a similar manner to that described previously [17]. The peptide epitopes and the tetra-branched LCP constructs were obtained in highly pure form without difficulties. The CD spectra were measured in water with trifluoroethanol (TFE) because this solvent is known to stabilize the α-helical structure in peptides and is often used to induce the native structure of protein fragments [22], [23], [24]. According to CD analysis peptide epitopes showed random coil rather than helical conformation with the minimum observed at 199 nm and 201 nm for peptides **1** and **2**, respectively, while LCP core alone clearly did not form helical structure (Figure 3). In contrast, **LCP-1** and **LCP-2** showed helical conformation, with double minima at 207 nm, 219 nm and 206 nm, 220 nm.

### II. Toxicological Evaluation

Basic toxicological evaluation of all compounds was performed using MTT and haemolytic assays (Figure 2). It was clearly demonstrated that all compounds were non-toxic to Caco-2 cells. Lipophilic compounds, **LCP-1** and **LCP-2** were also assessed for toxicity to red blood cells, and were shown to be non-haemolytic, even at high concentrations (100 µM).

### III. Antibody Binding and Neutralization of Recombinant *Na*-APR-1

**Figure 4:**
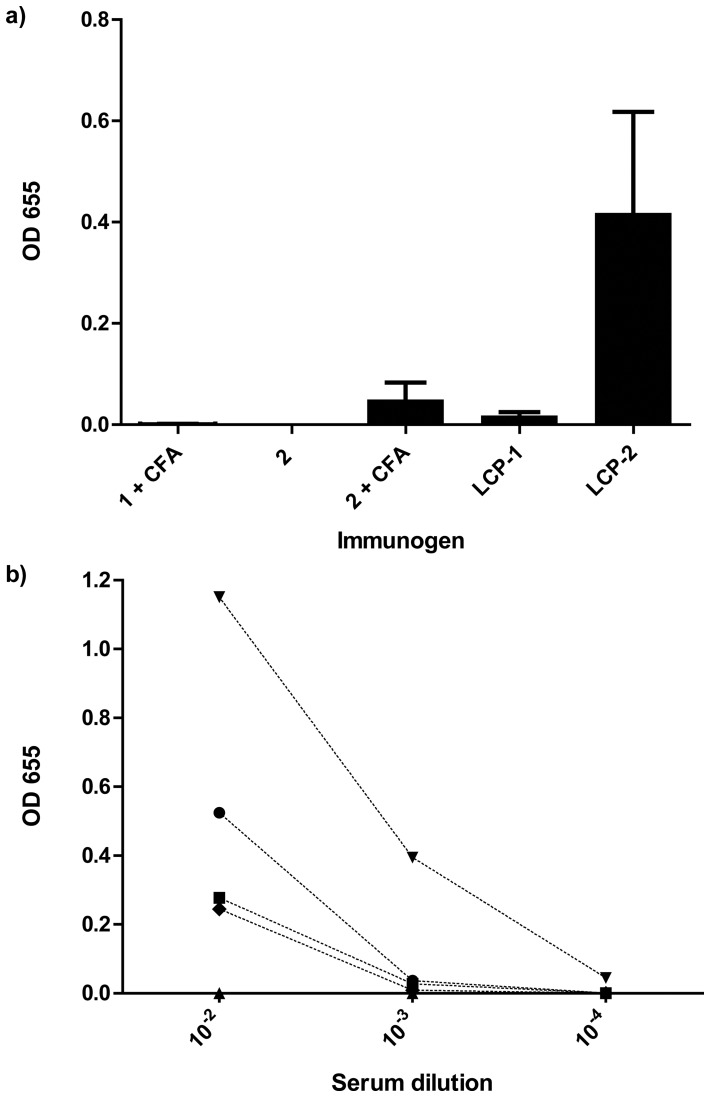
Comparison of antibody responses against peptides with adjuvant vs. LCP-peptides alone. (a) LCP-2 (A_291_Y-GCN4-LCP) is more immunogenic than peptide 2 (A_291_Y-GCN4 without LCP) formulated with Freund’s adjuvants. Antibody responses are depicted as OD values at a serum dilution of 1∶100 where each serum was used to probe its cognate immunogen peptide or LCP. (b) Higher antibody titres were observed in mice immunised with 60 µg of **LCP-2** without Freund’s adjuvants (experiment 2). Total IgG binding to plated **LCP-2** from individual mice on day 42 serial dilution from 1∶100 to 1∶10000. Each symbol represents a single animal.

Mice that were immunized with peptide **1** with Freund’s adjuvant did not induce a detectable IgG response in any mice. Peptide **2** did not induce an IgG response alone but did induce a weak IgG response when administered with adjuvant. **LCP-1** (A_291_Y-LCP) induced a very weak IgG response that was barely detectable, however **LCP-2** (A_291_Y-GCN4-LCP) induced a stronger IgG response but the titer was variable between individual mice (Figure 4a). We repeated the vaccination experiment with **LCP-2** (experiment 2) this time using 60 µg of LCP-4 in an attempt to improve the consistency and titer of the antibody response (Figure 4b). Increasing the quantity of **LCP-2** for immunization resulted in a more consistent antibody response between mice and higher titer responses.

IgG was purified from the two highest responding mice (Figure 4b) that received the 60 µg dose of **LCP-2** and used in an *Na*-APR-1 enzymatic neutralization assay. IgG (2.5 µg) from both mice inhibited the ability of APR-1 to digest a fluorescent peptide substrate by 92–100% compared to control IgGs. The inhibition is dose-dependent (Table 1) as described previously with polyclonal and monoclonal IgGs from mice immunized with recombinant *Na*-APR-1 [8], [11].

**Table 1:** Neutralisation of the enzymatic activity of recombinant *Na*-APR-1 using purified IgG from mice immunized with LCP-2.

IgG (µg)	RFU	% inhibition
**LCP-2 ms #1 (1.25)**	3021	57
**LCP-2 ms #1 (2.5)**	0	100
**LCP-2 ms #4 (1.25)**	3463	50
**LCP-2 ms #4 (2.5)**	312	92
**Control IgG (1.25)**	6978	–
**Control IgG (2.5)**	3983	–

Relative fluorescence units (RFU) are corrected to enzyme-free wells which contained substrate alone. Percent inhibition of enzymatic activity with each IgG was determined by establishing the baseline fluorescence using negative control IgG.

## Discussion

In our attempts to develop a human hookworm vaccine we identified a number of antigens that showed promise in animal models [6]. One of the lead antigens derived from the blood-feeding adult stage of the parasite was *Na-*APR-1 [8], [10]. There are numerous obstacles facing the production and safe use of a vaccine based on an active protease, so we identified an epitope from *Na*-APR-1, A_291_Y, that was capable of inducing antibodies that neutralized the catalytic activity of the recombinant enzyme [11]. Here we describe a self-adjuvanting approach to produce A_291_Y fused to GCN4 and LCP, and show that antibodies generated to the construct can neutralize the enzymatic activity of the native enzyme, highlighting the utility of this approach for the development of vaccines for neglected tropical diseases of humans.

The A_291_Y epitope was incorporated into the LCP system, previously shown to stimulate B-cell immune response by activation of dendritic cells via TLR-2 [25], [26]. In prior studies, we often observed difficulties in the synthesis and purification of branched LCP constructs and the use of conjugation techniques was necessary [18], [27], [28], [29]. In contrast, herein simple stepwise SPPS was applied for synthesis of all four compounds at high purity. The proper conformation of peptide epitopes is crucial for production of desired B-cell responses, thus we incorporated the A_291_Y epitope (AGPKAQVEAIQKY, **1**) in a coil-promoting sequence from the yeast GCN4 protein using a standard technique [15], [24] to induce native α-helical conformation of the A_291_Y epitope. Induction of helicity was not significant in the case of chimeric peptide **2**. However, when epitopes **1** and **2** were incorporated into LCP system, both (**LCP-1** and **LCP-2**) adopted more helical than random conformation (Figure 3). This was in agreement with our previous observation in the case of Group A *Streptococcus* vaccine candidates [16], [24], [28]. Not surprisingly, LCP-1 exhibited α-helical properties despite this conjugate not containing GCN4 flanking sequences. Induction of α-helicity of peptides due to their conjugation to dendritic LCP core was previously reported [28]. Similarly, helicity was also induced in peptides conjugated to other dendrimers, presumably due to dense packing of peptide epitopes in such structures [16]. **LCP-1** and **LCP-2** have amphiphilic properties (surfactant-like), therefore there was a risk that such compounds could be toxic to red blood cells. Using a standard haemolytic assay, the capacity of LCP compounds to induce haemolysis was examined and it was clearly demonstrated that these compounds were not haemolytic even at high concentration (Figure 2a). The preliminary toxicological studies were performed with Caco-2 cells using an MTT assay (Figure 2b). These human intestinal cells are often used to model toxicity of compounds *in vivo* [30], [31], [32], [33]. No considerable toxicity was observed for any of the tested compounds.

In two separate experiments, **LCP-2** induced inconsistent antibody responses in BALB/c mice, characterized by strong antibody responses in some mice and weak responses in others (Figure 4). Similar variability in responses was reported for immunization of mice with an LCP (J8) derived from a Group A *Streptococcus* vaccine candidate [34]. Despite the inconsistency in antibody responses, purified IgG directed against **LCP-2** neutralized the enzymatic activity of *Na*-APR-1, the primary goal of this study (Table 1). The ratio of antibody to protease required to neutralize the enzymatic activity was comparable to that obtained with anti-A_291_Y neutralizing monoclonal antibodies [11].

An ultimate human hookworm vaccine is likely to require at least two antigens, ideally from different developmental stages such as the infective third-stage larva (L3) and the adult worm, to achieve maximum efficacy [7]. *Na*-APR-1 is an ideal candidate antigen from the adult stage parasite, however it is not abundantly expressed in the L3. Antigens secreted by L3, such as *Na*-ASP-2 protect animals against heavy hookworm infections in pre-clinical studies in animals [35], but this antigen elicited an atopic response when administered to people in a hookworm-endemic area due to circulating IgE induced by natural exposure to hookworms [36]. Epitopes were identified in *Na*-ASP-2 that were unique to IgG and not IgE, implying that a peptide-based LCP vaccination strategy focusing on IgG-specific epitopes is a logical approach to developing human helminth vaccines. Such an approach might also integrate multiple epitopes from distinct antigens into the one LCP construct [27], [34], [37], as well as incorporation of universal T-helper epitopes [38], [39], [40] to improve consistency in antibody responses and to target outbred human populations. Overall, this work provides the first rational basis for development of the peptide-based vaccine against hookworm infection.
